# Investigating inequalities in patient outcomes for first-episode psychosis

**DOI:** 10.1192/bjp.2024.132

**Published:** 2024-12

**Authors:** Dasha Nicholls, Jobie Budd, Philippa Nunn, Paul French, Jo Smith, Veenu Gupta, Jonathan Holdship, Alan Quirk

**Affiliations:** Division of Psychiatry, Department of Brain Sciences, Imperial College London, London, UK; and Centre for Quality Improvement, Royal College of Psychiatrists, London, UK; Faculty, London, UK; and Division of Medicine, University College London, London, UK; Centre for Quality Improvement, Royal College of Psychiatrists, London, UK; Centre for Quality Improvement, Royal College of Psychiatrists, London, UK; Department of Research and Innovation, Pennine Care NHS Foundation Trust, Manchester, UK; and Department of Nursing and Public Health, Manchester Metropolitan University, Manchester, UK; Centre for Quality Improvement, Royal College of Psychiatrists, London, UK; and School of Allied Health and Social Care, University of Worcester, Worcester, UK; Centre for Quality Improvement, Royal College of Psychiatrists, London, UK; and Department of Psychology, Durham University, Durham, UK; Faculty, London, UK

**Keywords:** Early intervention in psychosis, ethnicity, first episode psychosis, inequalities, outcomes.

## Abstract

**Background:**

Understanding inequalities in outcomes between demographic groups is a necessary step in addressing them in clinical care. Inequalities in treatment uptake between demographic groups may explain disparities in outcomes in people with first-episode psychosis (FEP).

**Aims:**

To investigate disparities between broad demographic groups in symptomatic improvement in patients with FEP and their relationship to treatment uptake.

**Method:**

We used data from 6813 patients from the 2021–2022 National Clinical Audit of Psychosis data-set. Data were grouped by category type to obtain mean outcomes before adjustment to see whether disparities in outcomes remained after differences in treatment uptake had been accounted for. After matching, the average effect of each demographic variable in terms of outcome change was calculated. Moderator effects on specific treatments were investigated using interaction terms in a regression model.

**Results:**

Observational results showed that patients aged 18–24 years were less likely to improve in outcome, unless adjusted for intervention uptake. Patients classified as Black and Black British were less likely to improve in outcome (moderation effect 0.04, 95% CI 0–0.07) after adjusting for treatment take-up and demographic factors. Regression analysis showed the general positive effect of supported employment interventions in improving outcomes (coefficient −0.13, 95% CI −0.07 to −0.18, *P* < 0.001), and moderator analysis suggested targeting particular groups for interventions.

**Conclusions:**

Inequalities in treatment uptake and psychotic symptom outcome of FEP by social and demographic factors require monitoring over time. Our analysis provides a framework for monitoring health inequalities across national clinical audits in the UK.

Understanding the extent of inequalities in psychiatric health outcomes is a necessary first step in addressing them in clinical care. Socioeconomic and systemic factors, in addition to the effects of racism, xenophobia and discrimination, can have profound effects on health and access to care,^1^ inequalities that were exacerbated over the COVID-19 pandemic.^2^ This is of particular concern in psychosis,^3^ where early intervention is paramount.

Psychotic disorders can have devastating effects on quality of life and social functioning, resulting in stigmatisation, poor employment prospects and social exclusion. Early intervention in psychosis (EIP) services, specialised community-based multidisciplinary teams that work selectively with people in the early stages of a psychotic illness, deliver intensive treatment with an ‘assertive outreach’ model of care. Meta-analysis has shown superior outcomes for people who receive care from an EIP team compared with treatment as usual,^4^ and EIP services are thus cost-effective and widely implemented internationally. Physical health and premature mortality are extensively documented in this population and have been a recent focus for improvement.^5^ However, care that people with psychosis receive is not the only determinant of outcome. Psychosis disproportionally affects minority ethnic groups, and health inequalities influence access to services,^6^ as well as the type of care that is offered and taken up.^7^ Individuals from lower socioeconomic backgrounds experience greater symptom severity, longer duration of untreated psychosis and higher rates of relapse.^8–10^ They also face challenges accessing quality healthcare, delays in treatment initiation and discontinuation of services. Similarly, people from minority ethnic groups face barriers accessing culturally sensitive care, leading to treatment delays and poorer engagement with services.^11,12^ They often experience higher rates of involuntary hospital admission, longer hospital stays and greater symptom severity.^13–15^

Disparities in outcome following intensive early intervention for first-episode psychosis (FEP) have been less-well studied than access to, and take-up of, care. A systematic review of remission and recovery in FEP (82 cohorts, 18 randomised controlled trials) found no socioeconomic predictors associated with remission but did find that male gender and positive symptoms predicted recovery, although neither survived correction for multiple comparisons.^16^ Another review of 14 studies of heterogeneous quality and methodology found little or no evidence of disparities in outcome by ethnicity.^17^ However, recently Griffiths et al^18^ examined linear growth for positive, negative and general psychotic symptoms as well as functioning and depressive symptoms over a 5-year period in a sample of 978 people with FEP accessing EIP services, of whom 71 identified as Black and 157 as Asian. They found that ethnic group status accounted for variation in symptoms and function and that social deprivation, interacting with ethnicity, further contributed to this variance. The role of interventions was not examined.

The National Clinical Audit of Psychosis (NCAP) is one of 23 national clinical audits commissioned by England's Healthcare Quality Improvement Partnership (HQIP). NCAP collects anonymised clinical data from a random sample of patients with diagnosed or suspected FEP. The NCAP report recommends equitable access to treatment as part of a wider agenda to address health inequalities.^5^ The 2021–2022 NCAP audit contains case notes for 10 557 patients from all EIP service providers across England. Although NCAP has been running since 2017, the 2021–22 round is the first to have collected patient outcome metrics for positive psychotic symptoms (Health of the Nation Outcome Scale (HoNOS) item 6 – ‘Problems associated with hallucinations and delusions’^19^) at an initial and at a later follow-up stage.

Our study aimed to use demographic information and patient-level treatment and intervention take-up data to evaluate inequalities in psychotic symptom outcome, measured by HoNOS item 6, across broad demographic groups and by treatment take-up, and to investigate the effects of treatments in improving outcomes for these groups.

## Method

### Data-set

The data-set comprised patient-level data from the 2021–2022 EIP audit. Data were collected via a case-note audit and service-level questionnaire completed by EIP teams in England. National Health Service (NHS) trusts identified all eligible case notes and sent an anonymised list to the NCAP team, who selected a random sample of up to 100 patients per EIP team. Data were submitted from 10 557 case notes. Tabular pseudonymised patient-level data were made available that included variables denoting patient gender, age, ethnicity, employment and carer status, variables describing the take-up of a range of treatments and interventions, in addition to the outcomes of two assessments of HoNOS item 6, which refers to the presence of positive symptoms of psychosis in the form of hallucinations or delusions.^19^ Further information on this data-set and summary statistics are included in the NCAP audit report.^5^ Treatments include psychosis-focused cognitive–behavioural therapy (CBTp), psychosis-specific family interventions (conjoint with the identified patient), antipsychotic medication (with clozapine listed as separate variable), supported employment and carer-only interventions. Physical health intervention variables included the provision of interventions for smoking, alcohol, other substance misuse, weight gain, hypertension, diabetes and dyslipidaemia. Additional variables on screening and suitability of physical interventions were not included in this analysis.

### Demographic variables

The NCAP data-set contains variables for patient age, gender and ethnicity. For this study, age was divided into categories (18–24, 25–34, 35–44, 45–54, 55–69 years) to limit discrete combinations in interaction analysis and for consistency. The few patients over the age of 64 were grouped with those aged 55 and over. The NCAP data-set provides gender categories of ‘Female’, ‘Male’ and ‘Non-binary/other’. Office for National Statistics (ONS) ethnic groups (Asian or Asian British, Black or Black British, Mixed ethnicity, Other ethnicity, and White) were used rather than more granular ethnic categories to ensure that counts were sufficient for statistical analysis.

### Study population and inclusion criteria

**Fig. 1:**
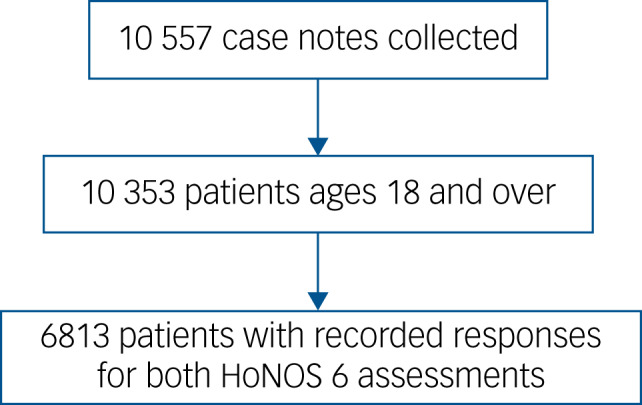
Flowchart showing stages of study exclusion and cohort size at each stage. HoNOS 6, item 6 on the Health of the Nation Outcome Scale.

The study population included patient case notes from the 2021–2022 EIP audit where patients were aged 18 and over and had both primary and secondary HoNOS item 6 assessments recorded. Figure 1 shows the cohort size at each stage of exclusion. Baseline distributions of patient gender and broad ethnicity category were compared with those of the filtered data-set after exclusions using a χ^2^-test, to ensure that no new demographic biases were introduced. Likewise, the distribution of age groups in the data-set including all patients aged 18 and over was compared with the filtered data-set after exclusions using a χ^2^-test. Significance was set to an alpha level of 0.05 and all *P*-values were two-sided. No significant differences were observed.

The filtered data-set cohort of 6813 patients had a mean age of 30 years, a minimum age of 18 and a maximum age of 67; 38.87% were listed as female; and 13.15% were listed as belonging to the Asian or Asian British ethnicity category, 13.31% Black or Black British, 3.85% Mixed ethnicity, 64.17% White and 3.08% belonging to another ethnicity category.

### Statistical analysis

#### Observational outcome change

The filtered data-set was grouped by each demographic category type individually to obtain counts and mean assessment outcomes by demographic category. Negative outcome changes indicated an improvement in psychosis symptoms (i.e. reduction in positive symptoms). One thousand bootstrap samples were grouped and aggregated to obtain the ranges of 95% confidence intervals of the mean outcome change between the two assessments.

We undertook adjustment to see whether disparities in outcomes remained after differences in the uptake of treatment has been accounted for, in order to identify any inequalities in the effectiveness of treatments. Change in outcome is also heavily dependent on initial HoNOS item 6 score, where the distribution of outcome change differs by each initial assessment score.

#### Matching

**Fig. 2:**
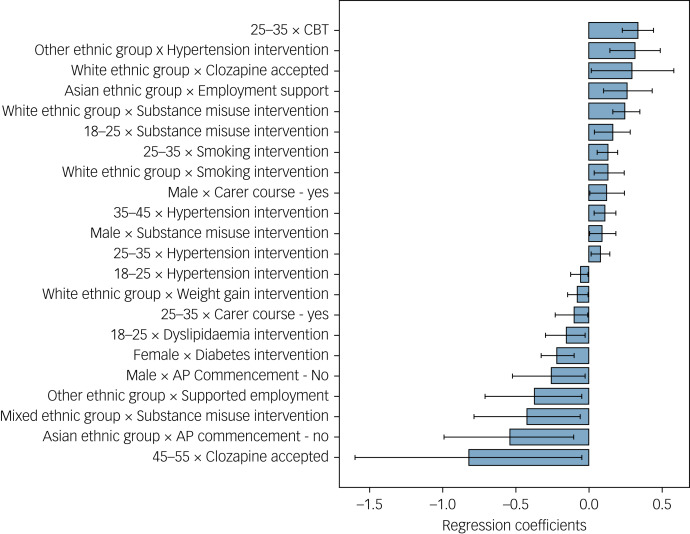
Statistically significant interaction effects, here shown with regression coefficients. Negative coefficients show interactions with an improvement in the severity of psychosis symptoms. CBT, cognitive–behavioural therapy; AP, antipsychotic medication.

To report the association of demographic factors and differences in outcomes, adjustments were required to control for the effects of confounding variables and selection bias in the observational EIP audit data-set. Matching, a causal inference technique,^20^ was chosen as an appropriate adjustment method, because of the imbalanced number of patients with each outcome score, the non-linear relationship between treatment uptake and outcomes, and presence of multiple demographic and treatment variables. A full causal graph of data-set variables is presented in Supplementary Fig. 1, available at https://doi.org/10.1192/bjp.2024.132, together with a justification for including a reduced data-set of variables given in the text and Fig. 2. Each patient was matched to a single k-nearest neighbour,^21^ based on binary treatment and intervention take-up variables (taken up versus not offered/not eligible/refused) and encoded demographic categories (excluding the demographic variable being inspected, for example if the effect of patients being in the 18–24 age group was being inspected, patients were matched only on the gender and ethnicity demographic variables). This matching process resulted in resampling patients because not all matches were unique. Once an adjusted data-set was produced by matching on each demographic category, the average effect of each demographic variable in terms of outcome change was calculated. The range of 95% confidence intervals was obtained from 1000 bootstrap samples. All analyses were performed in Python (version 3.9 for Linux; https://repo.anaconda.com/miniconda/).

#### Moderator interaction effects

Moderator effects on specific treatments were investigated using interaction terms in a regression model as suggested by Breitborde et al.^22^ To determine whether specific demographic factors moderated (influenced) the effectiveness of treatments, interaction terms (the product of encoded category variables) were created and their significance in affecting outcomes were tested in a regression model. A probit ordinal regression model (Python statsmodels) was used to predict the ordered categories of numerical change in HoNOS item 6 score between initial and follow-up assessments. Features included initial assessment outcome, binary treatment and physical health intervention take-up variables (taken up versus not offered/not eligible/refused), gender, age group and broad ethnicity category, in addition to interaction terms (the product of binary demographic and treatment/intervention variables). If the interaction term is a statistically significant predictor (statsmodels calculates *P*-values on the basis of *z*-statistics), it suggests that the demographic variable influences the relationship between the treatment or intervention variable and change in outcome. All analyses were performed in Python.

## Results

Table 1 shows outcome changes by demographic group. Between the initial and follow-up assessments, all groups improved in terms of psychosis symptom severity as measured by HoNOS item 6. There are two key demographic effects. First, males improved less, on average, than females; and second, patients aged 18–24 improved less than other age groups.

**Table 1:** Outcomes by broad demographic category

Demographic category type	Demographic category	Sample size, *n*	Initial outcome mean	Follow-up outcome mean	Mean outcome change (95% CI)
Gender	Female	2648	2.74	2.03	−0.72 (−0.77 to −0.66)
	Male	4154	2.89	2.21	−0.68 (−0.72 to −0.64)
	‘Other/non-binary’	11	2.64	2.09	−0.55 (−1.27 to 0.18)
Age group	18–24	1914	2.84	2.17	−0.67 (−0.73 to −0.6)
	25–34	2410	2.82	2.14	−0.68 (−0.74 to −0.63)
	35–44	1194	2.86	2.13	−0.73 (−0.81 to −0.65)
	45–54	758	2.81	2.16	−0.66 (−0.75 to −0.56)
	55–69	537	2.10	2.03	−0.77 (−0.88 to −0.65)
Broad ethnic category	Asian or Asian British	896	2.83	2.08	−0.75 (−0.85 to −0.66)
	Black or Black British	907	2.78	2.11	−0.67 (−0.77 to −0.57)
	Mixed	262	2.79	2.05	−0.74 (−0.91 to −0.58)
	White	4372	2.84	2.16	−0.68 (−0.71 to −0.64)
	Other ethnicity	210	2.94	2.19	−0.76 (−0.96 to −0.55)
	Refused	15	2.93	2.53	−0.39 (−1.13 to 0.27)
	Unknown/undocumented	151	2.89	2.10	−0.79 (−1.05 to −0.55)

Table 2 shows outcome changes by treatment take-up. Patients not offered CBTp improved less, on average, in terms of psychosis symptom severity than those who refused, were waiting for or took up CBTp.

**Table 2:** Outcomes by treatment take-up

Treatment type	Treatment category	Sample size, *n*	Initial outcome mean	Follow-up outcome mean	Mean outcome change (95% CI)
CBTp	Took up	3315	2.85	2.15	−0.7 (−0.75 to −0.66)
	Not offered	793	2.74	2.16	−0.58 (−0.68 to −0.48)
	Refused	2071	2.83	2.12	−0.72 (−0.78 to −0.65)
	Waiting	634	2.86	2.15	−0.71 (−0.82 to −0.59)
Family therapy	Took up	1486	2.81	2.14	−0.68 (−0.74 to −0.61)
	Not offered	1890	2.87	2.20	−0.67 (−0.73 to −0.61)
	Refused	3081	2.81	2.09	−0.72 (−0.77 to −0.67)
	Waiting	356	2.87	2.23	−0.63 (−0.78 to −0.48)
Supported employment	Took up	2411	2.78	2.05	−0.73 (−0.79 to −0.67)
	Not offered	1818	2.88	2.17	−0.71 (−0.77 to −0.64)
	Refused	2351	2.83	2.20	−0.63 (−0.69 to −0.58)
	Waiting	233	2.99	2.17	−0.82 (−1.01 to −0.65)
Antipsychotic treatment commencement	>12 months ago	5761	2.80	2.12	−0.68 (−0.71 to −0.64)
	6–12 months ago	562	3.18	2.33	−0.85 (−0.96 to −0.72)
	<6 months ago	150	2.87	2.34	−0.52 (−0.75 to −0.31)
	No	340	2.80	2.12	−0.76 (−0.89 to −0.63)
Clozapine	Took up	243	3.37	2.95	−0.42 (−0.58 to −0.26)
	Not offered	314	3.03	2.67	−0.36 (−0.53 to −0.21)
	Refused	141	3.45	3.09	−0.36 (−0.57 to −0.16)
	No data (not eligible)	6115	2.79	2.06	−0.73 (−0.76 to −0.69)
Carer course	Took up	2809	2.83	2.18	−0.66 (−0.71 to −0.61)
	No	2550	2.78	2.07	−0.71 (−0.76 to −0.65)
	No data (not eligible)	1454	2.92	2.18	−0.74 (−0.81 to −0.67)

CBTp, psychosis-focused cognitive–behavioural therapy.

Table 3 lists the average moderating effect of belonging to demographic groups on outcomes, first when adjusted by differences in initial assessment score, and additionally when adjusted by differences in initial assessment score, treatment and intervention take-up, and demographic variables using the matching to a nearest neighbour method. Patients in the ‘Non-binary/other’ gender category and in the ‘Refused’ or ‘Unknown/undocumented’ ethnicity categories remain in the study data-set for this analysis but summary statistics are not reported owing to low counts. Matching only on initial outcome showed being in the 18–24 age group had a negative effect on outcomes compared with other age groups. However, when matched on all variables, including treatments, this effect was no longer present. Table 3 shows that treatments are less likely to be effective for male patients, patients in the Black and Black British ethnicity category, and patients aged 25–34.

**Table 3:** Average moderation effect of demographic groups on outcomes

Demographic category type	Demographic category	Matching on initial outcome (95% CI)	Matching on initial outcome, treatment and intervention take-up, and demographics (95% CI)
Gender	Female	−0.42 (−0.39 to −0.45)	−0.16 (−0.12 to −0.19)
	Male	**0.54** (**0.57 to 0.51)**	**0.16** (**0.2 to 0.12)**
Broad ethnic category	Asian or Asian British	0.01 (0.04 to −0.02)	−0.01 (0.03 to −0.04)
	Black or Black British	−0.02 (0.01 to −0.05)	**0.04** (**0.07 to 0)**
	Mixed	−0.4 (−0.36 to −0.44)	−0.03 (0 to −0.07)
	Other ethnicity	−0.67 (−0.63 to −0.7)	0.01 (0.05 to −0.03)
	White	−0.01 (0.02 to −0.04)	0.03 (0.06 to −0.01)
Age group, years	18–24	0.63 (0.66 to 0.6)	−0.01 (0.02 to −0.05)
	25–34	**0.23** (**0.26 to 0.2)**	**0.06** (**0.1 to 0.03)**
	35–44	−0.17 (−0.14 to −0.21)	−0.02 (0.01 to −0.06)
	45–54	**0.33** (**0.37 to 0.3)**	−0.04 (−0.01 to −0.08)
	55–69	**0.19** (**0.22 to 0.16)**	−0.13 (−0.09 to −0.16)

Worse (positive change in) outcomes are indicated in bold.

The interaction analysis summarised in Fig. 2 attempts to investigate for what specific treatments and demographic groups demographic factors may have an impact on outcome. The coefficients and *P*-values of variables in an ordinal probit model when interaction variables are not included can be found in Supplementary Table 1. Supported employment interventions seem to have a particularly positive effect on patient outcomes (coefficient −0.13, 95% CI −0.07 to −0.18, *P* < 0.001), with additional positive effects for weight gain interventions where offered (coefficient 0.11, 95% CI −0.05 to −0.17, *P* < 0.001).

## Discussion

To our knowledge, this is the first study to examine disparities in outcome from FEP in a large national data-set and to explore intersectionality between demographic factors and intervention. We used a statistical framework similar to that used by Freitas et al^23^ to monitor inequalities in involuntary admission under the Mental Health Act in London. Overall, patient outcomes improved significantly in terms of reduction in psychotic symptoms (hallucinations and delusions) over time across the sample in all broad demographic categories (age, gender, ethnicity) except where the sample was too small to confirm significance. Outcomes also improved across treatment groups, including where treatment was refused, not offered or waiting. De Nijs et al^24^ likewise used demographic and baseline patient characteristics to predict outcomes for psychosis with over 60% accuracy based on factors such as Global Assessment of Functioning (GAF) scores, psychotic symptoms, quality of life, antipsychotic use, psychosocial needs and depressive symptoms.

Our results suggest that demographic factors have some overall effect on treatment outcomes. We found an effect of age on outcome, patients in the younger age group^18–24^ having worse outcome than those who were older. However, once adjusted for other variables, including treatment, this group had slightly better outcomes. Effectively, patients aged 18–24 could be said to have worse outcomes because they take up treatment less often, and when treatment is taken up, they do as well as other age groups in terms of symptom improvement. In a recent systematic review of clinical recovery in people with FEP, based on 26 unique study samples including 3877 individuals (mean age 26.4 years), none of the variables examined predicted recovery.^25^ These included age at inclusion (*P* = 0.84). Clinical recovery was defined in different ways for each study, varying from no hospital admissions to various measures of function, a common definition being a GAF score of 60 or more. Our study examined improvement in psychotic symptoms only, and not recovery, which may account for our positive finding.

Even when matched on baseline HoNOS 6 score, treatment offer and take-up, and demographics, we found that men, people identified as Black or Black British, and people aged 25–34 improved less than other people. Previous research about the role of gender in psychosis outcome have been mixed, but a systematic review of treatment-resistant schizophrenia (12 studies, 6 of which were high quality, 11 958 participants) found men were 1.57 times more likely to be considered ‘treatment resistant’ than women (95% CI 1.11–2.21, *P* = 0.010), and that around 22.8–24.4% of FEP cohorts would be considered ‘treatment resistant’. These findings align with our data showing a difference in outcome by gender as well as an interaction between gender and treatment. This is an important finding that has clinical implications and suggests that understanding barriers to treatment take-up among young men should be a research priority. Indeed, studies showing gender differences at entry, take-up and outcomes have concluded that gender-specific therapeutic strategies should be considered in early intervention services.^26,27^ The NCAP report showed that men are more likely to be offered clozapine than women and women are more likely to engage with CBTp than men. Together these suggest that men, especially young men, are less likely to take up interventions or respond to antipsychotic treatment and this largely, but not entirely, accounts for the variance in outcome by gender.

Our findings on variation in outcome by patient ethnicity align with and build on the recent study by Griffiths et al, which found an influence of ethnicity and an interaction with socioeconomic status on illness trajectories and these factors also determined the likelihood of needing continued care after discharge from EIP services.^18^ There are a number of factors that might account for the differential outcomes that our study confirms. First, individuals from ethnic minorities may face cultural and language barriers that hinder access to appropriate mental healthcare, leading to delays in help-seeking and a higher likelihood of reaching care later. We were unable to examine the effects of untreated illness on outcome, although the Griffiths et al study^18^ found that those in the Black ethnic group had a shorter, not a longer, duration of untreated illness. Second, mental health services may not adequately address the unique cultural and social needs of minority ethnic populations. This can contribute to misdiagnosis, inadequate treatment or lack of engagement with services. However, our methodology enabled us to parse out the effects of ethnicity independent of treatment uptake. Socioeconomic disadvantage, which shows intersectionality with ethnicity, may influence access to quality community-based support, including opportunities for supported employment and family support, merits further investigation as a factor that may contribute to this disparity in outcome by ethnic group. Other findings of intersectionality on poorer outcome in our data included White ethnicity and take-up of clozapine, which could be accounted for by illness severity since clozapine is only offered to those who are non-responders to two antipsychotics; and Asian or White Asian ethnicity and take-up of supported employment. A recent study of patient-reported outcomes from NCAP found that participants were more likely to report that their mental health had improved if they had been offered CBTp or targeted interventions for carers, and that participants from Black/Black British ethnic groups were more likely to recommend their care than those from White ethnic groups.^28^ This contrasts with our finding of inequalities in symptom remission (i.e. change in clinician-reported outcomes), highlighting the differences between symptom remission and experience of and satisfaction with an EIP service. However, other recent NCAP analyses found that every minoritised ethnic group, except those of Mixed Asian–White and Mixed Black African–White ethnicities, had lower adjusted odds of receiving CBTp and many also experienced lower adjusted odds of receiving family intervention,^29^ both of which were important for experience and outcome. Together these findings emphasise the importance of exploring cultural, economic and social factors when offering interventions to support recovery if inequalities in outcome by ethnicity are to be minimised. For example, culturally adapted psychological interventions are more efficacious in comparison with treatment as usual, proportionate to the degree of adaptation.^30^

Findings were mixed in our sample regarding the interaction of demographic factors and physical health interventions, including interventions for hypertension and for substance misuse. Again, these are only offered to a limited number (those who screen positive for risk), although our design matched for these factors. A systematic review and random-effects meta-analysis of prospective and retrospective, nationwide and targeted cohort studies assessing moderators of mortality risk in people with schizophrenia compared with the general population (sample with schizophrenia: *n* = 4 536 447 from 135 studies) found that all-cause mortality was increased in people with schizophrenia, with the largest risk in first-episode (relative risk = 7.43, 95% CI 4.02–13.75, *n* = 2) and incident (i.e. earlier-phase) schizophrenia (relative risk = 3.52, 95% CI 3.09–4.00, *n* = 7) compared with the general population.^31^ Comorbid substance use disorder increased all-cause mortality (relative risk = 1.62, 95% CI 1.47–1.80, *n* = 3) and antipsychotics were protective against all-cause mortality compared with no antipsychotic use (relative risk = 0.71, 95% CI 0.59–0.84, *n* = 11), with large effects. Variation by ethnicity in these outcomes was not included, suggesting that further research is needed in this area.

### Limitations

The audit did not record information on the date of each outcome assessment, meaning that time between outcomes could range between 0 and 365 days. Consequently, the analysis and subsequent results assume uniform time between assessments and the outcome changes of individual patients are not appropriately weighted. The effect of a longer or shorter assessment interval than the population mean for certain services or demographic groups, affecting the recorded efficacy of their treatment, may potentially bias our understanding of treatment effectiveness in those groups. The effect of this cannot be known unless the interval is recorded. Our recommendation for future data collection/processing for analysis is that the date of outcome measurement is included in the data-set.

The choice of psychometric measure (HoNOS item 6) for this study was driven by data availability. However, HoNOS item 6 has imperfect interrater reliability, and it is undocumented whether a different clinician made each assessment.^32^ Future research should consider outcomes such as clinical impairment as well as psychosis symptomatology.

The use of broad gender and demographic categories, while necessary to reduce statistical uncertainty, make the results potentially less useful for targeted monitoring and intervention in clinical practice. The failure of the ONS categories to capture some ethnicity categories (e.g. Latinx), and the small non-binary gender sample mean that analysis for these groups that may have specific needs are not possible. Greater specificity in ethnicity coding is a priority if inequalities in healthcare are to be properly understood.

Some of the treatment variables are conditional in terms of being appropriate or offered. For example, employment support is only offered to those who are unemployed, and clozapine is only prescribed to those who have not responded to other antipsychotic medication.

Although there is known intersectionality between socioeconomic status and ethnicity, we had no measure of socioeconomic status, and geographical information such as local authority and national region were not available. Consequently, reported effects of demographic factors cannot be separated from the effects of location (for instance, we do not know whether younger patients improve less in psychosis symptom severity because patients in urban areas improve less, since the population in urban areas is younger). It was also not possible to extract variables such as local area deprivation index, or local access to services or green space.

Several important variables relevant for FEP outcomes were not measured and therefore not in the analysis, including diagnostic breakdown (e.g. schizophrenia versus brief psychotic disorder/delusional disorder), duration of untreated psychosis, age at onset and comorbidity. Future analyses should consider the impact of substance use (HoNOS item 3) on outcomes.

### Conclusions

Addressing health inequalities in first-episode psychosis requires multifaceted approaches. The persistence of disparities after adjusting for differing rates of treatment take-up suggests a significant effect of external factors that require further monitoring. Interventions should focus on improving access to early intervention services, ensuring culturally sensitive care, reducing treatment delays and providing comprehensive support tailored to the needs of disadvantaged populations. Further research is needed to explore the specific mechanisms underlying these disparities and develop targeted interventions to reduce health inequalities in this population. We propose our analysis as a framework for monitoring health disparities across the many national clinical audits in the UK.

## Supporting information

Nicholls et al. supplementary material Nicholls et al. supplementary material

## Data Availability

The data that support the findings of this study are openly available on application to the Data Access Request Group at the Healthcare Quality Improvement Partnership (HQIP) (https://www.hqip.org.uk/national-programmes/accessing-ncapop-data/) or applicants can email DataSharing@hqip.org.uk before a submitting a formal application.
