# Supplementary material for: Investigating inequalities in patient outcomes for first-episode psychosis
Source: Br J Psychiatry. 2024 Dec;225(6):556–62. doi: 10.1192/bjp.2024.132 (PMC11669469; doi:10.1192/bjp.2024.132)
Supplement: Nicholls et al. supplementary material [file S0007125024001326sup001.docx]

**Supplementary Information**


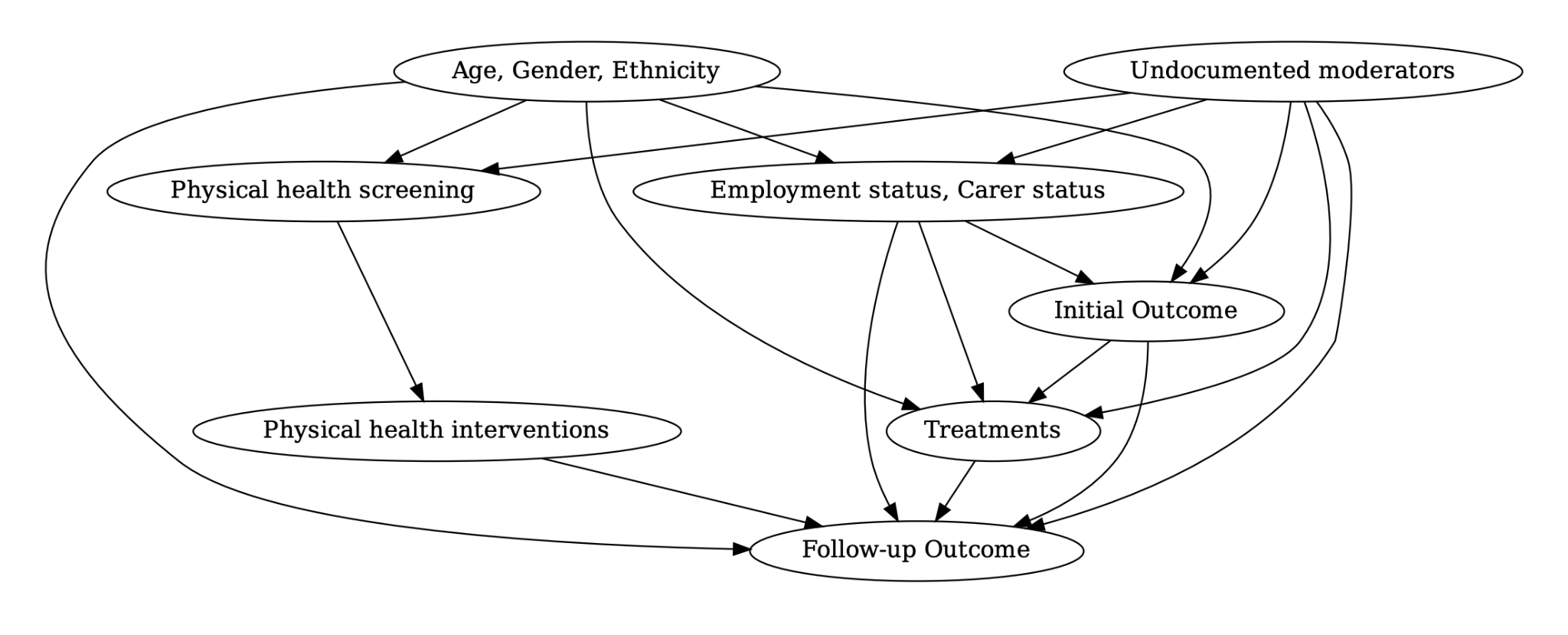


**Supplementary Figure 1.** Full causal graph of dataset variables


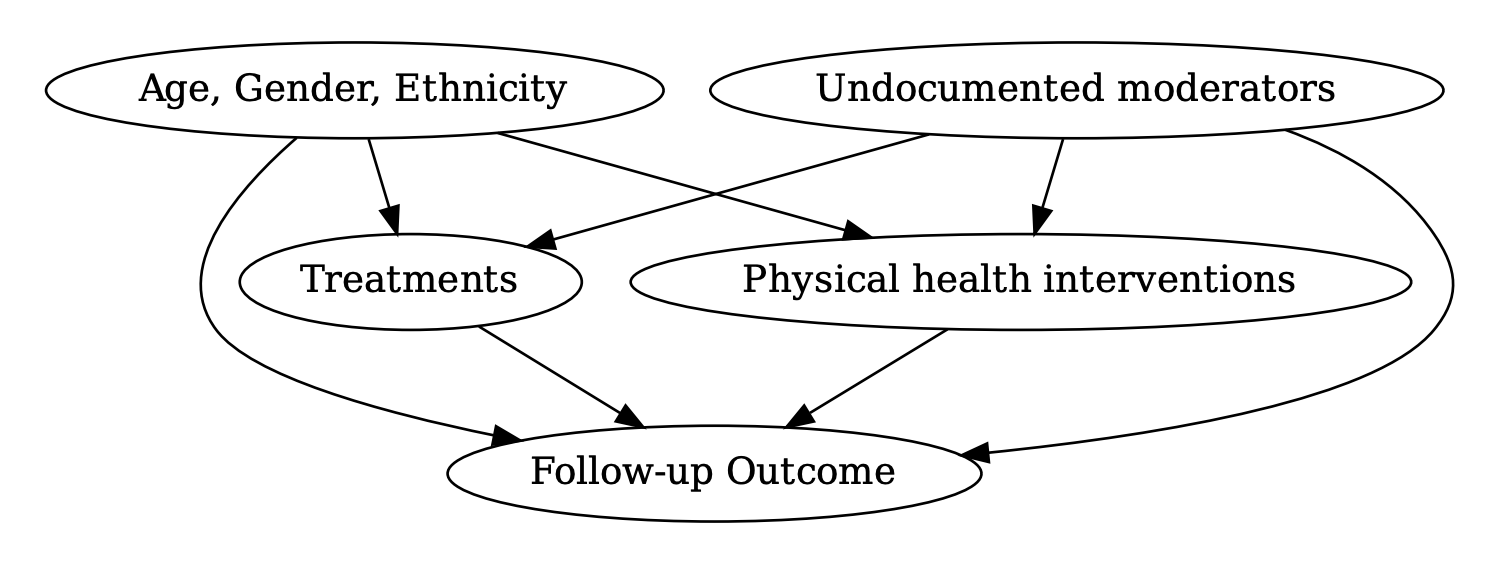


**Supplementary Figure 2.** Reduced causal graph of dataset variables

Justification for variable inclusion/exclusion

The full causal graph of dataset variables is shown in **Supplementary Figure 1.** Moderator variables, documented (age, gender and ethnicity) and undocumented, are fixed and not affected by the other variables. Moderator variables can be said to affect outcomes directly, and indirectly via their influence on other variables that also affect outcomes.

Follow-up outcomes are heavily influenced by initial outcomes. We can remove this from our graph by looking at either follow-up outcomes given each initial outcome separately, or by looking at change in outcome and leaving initial outcome as a variable (as change in outcome dependent on initial outcome).

Employment status and carer status are influenced by the moderator variables, and will directly influence which treatments are offered (such as supported employment and carer interventions), indirectly influencing follow-up outcomes. This could introduce selection bias, so we did not include these variables in our model.

Physical health interventions are only provided if physical health screening has been provided and if patients' physical health meets a threshold where intervention is required. e.g. if smoker status is screened, and if the patient smokes, then a smoking intervention will be offered and may be taken up. Since the variable listing whether interventions were taken up includes information about screening being offered and taken up, and about the patients' physical health, we can just include this variable. The reduced causal graph of dataset variables is shown in **Supplementary Figure 2.**

**Supplementary Table 1.** Coefficients of variables in an ordinal model predicting change in outcome (not including interaction variables)

|  | coef | p-value | lower | upper |
| --- | --- | --- | --- | --- |
| Q6a_Item_6 | 0.36 | 0.00 | 0.34 | 0.38 |
| Q7_CBTp_Tookup | 0.03 | 0.30 | -0.03 | 0.08 |
| Q7_Family_Intervention_Tookup | -0.07 | 0.05 | -0.14 | 0.00 |
| Q7_Supported_employment_Tookup | -0.13 | 0.00 | -0.18 | -0.07 |
| Q8_AP_commencement_No | -0.03 | 0.64 | -0.15 | 0.09 |
| Q9a_Offer_Clozapine_Yes, the person accepted clozapine | 0.19 | 0.01 | 0.05 | 0.33 |
| Q10_Carer_Course_Yes | 0.08 | 0.00 | 0.02 | 0.14 |
| Q1_Gender_Female | -0.21 | 0.56 | -0.89 | 0.48 |
| Q1_Gender_Male | -0.07 | 0.83 | -0.76 | 0.61 |
| Q3_Ethnicity_upper_AsianorAsianBritish | 0.10 | 0.29 | -0.08 | 0.29 |
| Q3_Ethnicity_upper_BlackorBlackBritish | -0.12 | 0.19 | -0.31 | 0.06 |
| Q3_Ethnicity_upper_Mixed | -0.08 | 0.48 | -0.30 | 0.14 |
| Q3_Ethnicity_upper_Otherethnicgroups | -0.08 | 0.49 | -0.31 | 0.15 |
| Q3_Ethnicity_upper_White | 0.04 | 0.66 | -0.13 | 0.21 |
| age_group_[18,25) | 0.07 | 0.20 | -0.04 | 0.19 |
| age_group_[25,35) | 0.04 | 0.42 | -0.06 | 0.15 |
| age_group_[35,45) | 0.02 | 0.79 | -0.10 | 0.13 |
| age_group_[45,55) | 0.02 | 0.79 | -0.11 | 0.14 |
| S7_Smoking_intervention_provided | 0.04 | 0.24 | -0.03 | 0.10 |
| S7_Alcohol_intervention_provided | -0.08 | 0.05 | -0.17 | 0.00 |
| S7_SubstanceMisuse_intervention_provided | 0.08 | 0.04 | 0.00 | 0.16 |
| S7_WeightGain_intervention_provided | -0.11 | 0.00 | -0.17 | -0.05 |
| S7_Hypertension_intervention_provided | 0.01 | 0.54 | -0.02 | 0.05 |
| S7_Diabetes_intervention_provided | -0.03 | 0.43 | -0.11 | 0.05 |
| S7_Dyslipidaemia_intervention_provided | 0.07 | 0.05 | 0.00 | 0.15 |
| Initial HONOS item 6 score of 1.0 - 2.0 | 0.73 | 0.05 | 0.01 | 1.45 |
| Initial HONOS item 6 score of 2.0 - 3.0 | -0.66 | 0.00 | -0.71 | -0.61 |
| Initial HONOS item 6 score of 3.0 - 4.0 | -0.24 | 0.00 | -0.29 | -0.20 |
| Initial HONOS item 6 score of 4.0 - 5.0 | 0.03 | 0.43 | -0.04 | 0.09 |
